# Computer-aided identification of polymorphism sets diagnostic for groups of bacterial and viral genetic variants

**DOI:** 10.1186/1471-2105-8-278

**Published:** 2007-08-01

**Authors:** Erin P Price, John Inman-Bamber, Venugopal Thiruvenkataswamy, Flavia Huygens, Philip M Giffard

**Affiliations:** 1 Cooperative Research Centre for Diagnostics, Institute of Health and Biomedical Innovation, Queensland University of Technology, Cnr Blamey St and Musk Ave, Kelvin Grove, Australia

## Abstract

**Background:**

Single nucleotide polymorphisms (SNPs) and genes that exhibit presence/absence variation have provided informative marker sets for bacterial and viral genotyping. Identification of marker sets optimised for these purposes has been based on maximal generalized discriminatory power as measured by Simpson's Index of Diversity, or on the ability to identify specific variants. Here we describe the Not-N algorithm, which is designed to identify small sets of genetic markers diagnostic for user-specified subsets of known genetic variants. The algorithm does not treat the user-specified subset and the remaining genetic variants equally. Rather Not-N analysis is designed to underpin assays that provide 0% false negatives, which is very important for e.g. diagnostic procedures for clinically significant subgroups within microbial species.

**Results:**

The Not-N algorithm has been incorporated into the "Minimum SNPs" computer program and used to derive genetic markers diagnostic for multilocus sequence typing-defined clonal complexes, hepatitis C virus (HCV) subtypes, and phylogenetic clades defined by comparative genome hybridization (CGH) data for *Campylobacter jejuni*, *Yersinia enterocolitica *and *Clostridium difficile*.

**Conclusion:**

Not-N analysis is effective for identifying small sets of genetic markers diagnostic for microbial sub-groups. The best results to date have been obtained with CGH data from several bacterial species, and HCV sequence data.

## Background

The last two decades have seen an exponential increase in the generation of comparative genetic data from within bacterial and viral species. Many of the bacterial data sets are derived from electrophoresis-based genotyping methods, such as pulsed-field gel electrophoresis, which has been used to develop the inter-laboratory PulseNet system for real-time monitoring of foodborne bacterial pathogens [1]. More recently, databases of defined genetic polymorphisms have become available. Conspicuous examples are multilocus sequence typing (MLST) databases [2,3], the results of comparative genome hybridization (CGH) studies on bacteria [4-7], and whole-genome sequence databases for bacteria and viruses [8-12].

The extensive knowledge base of comparative genetic information can be exploited to develop rationally-designed genotyping methods for examining epidemiology, or inferring virulence potential, vaccine susceptibility or antimicrobial-antiviral resistance. One approach to discriminating known genotypes within a species is to interrogate every known genetic polymorphism. However, this approach is inefficient due to linkage of alleles, and may also provide more resolving power than is required [13]. Despite considerable improvements in nucleic acid analysis technology in recent years, there remains a need for cost-effective and rapid genotyping methods that interrogate small sets of polymorphisms and provide the required information in an efficient manner [14]. Such methods have potential applications in infection control, point-of-care diagnosis, high-throughput public health investigations, food microbiology and biodefense. Suitable emerging technology platforms for such marker sets include real-time PCR [15], medium-density arrays [16], and more recently 'lab-on-a-chip' devices [17,18].

The considerable volume of comparative genetic information now available renders computerized data-mining the only practical means of identifying sets of polymorphisms optimized for particular genotyping tasks. Our research group has previously developed and described the "Minimum SNPs" computer program, which extracts resolution-optimized single-nucleotide polymorphism (SNP) sets from complex DNA sequence alignments [19]. Previously described capabilities of "Minimum SNPs" included the identification of SNP sets that discriminated a single sequence variant from all other know variants (the '%' mode), and the identification of SNP sets that maximize Simpson's Index of Diversity (*D*), and are therefore optimized with respect to discriminating all variants from each other [19,20]. The % module has been applied to the identification of highly informative SNPs that define specific *Neisseria meningitidis *and *Staphylococcus aureus *variants [19], while the *D *module has been used to extract *D*-maximized SNP sets from *S. aureus *and *Campylobacter jejuni *MLST databases [21-23], and, more recently, to derive *D*-maximized binary gene sets (sets of genes that are present in some isolates but not others) from *C. jejuni *CGH data [24].

Other bioinformatics programs that carry out similar functions to "Minimum SNPs" include a linkage disequilibrium-selection algorithm, which identifies SNPs diagnostic for haplotype blocks in mammalian genomes [13], and SNPT, which also incorporates a *D *maximization module [25]. One function notably absent from previous versions of "Minimum SNPs" and similar programs is the ability to identify sets of genetic markers that discriminate a user-defined group of variants from all other known variants. Such a function could underpin genotyping methodologies designed to identify all variants within a species that possess specific traits of interest, such as increased virulence or resistance properties. There are several considerations when designing such an algorithm, such as conversion of the user-defined group of sequence variants into a consensus sequence, and scoring of the resolving power of genetic marker sets. Here we report a novel algorithm for identifying such genetic marker sets, and its application to the analysis of microbial and viral comparative genetic data.

## Results and Discussion

### MLST datasets

The clonal complexes (CCs) defined by MLST data are emerging as important epidemiological or taxonomic units [14,26]. Therefore it was investigated whether Not-N analysis could identify diagnostic SNPs for major CCs of a variety of bacterial species. In *E. coli*, two independent MLST schemes were examined. Using scheme 1, Not-N analysis successfully identified 15 SNPs that completely differentiated the 10 major CCs (see additional file 1). The second *E. coli *MLST scheme, which contains a larger cohort of isolates than scheme 1, required 24 SNPs to differentiate the 12 major CCs. Not-N analysis was unable to completely differentiate the largest CC, with eight SNPs resolving 98.5% of the out-group from the group of interest. Not-N analysis of the *H. influenzae *MLST dataset identified 24 SNPs that differentiated the seven major CCs from the remaining ST population (results not shown).

In contrast to *H. influenzae *and *E. coli*, Not-N analysis was unable to identify high-confidence SNPs (≥ 98%) for four *S. aureus *and five *C. jejuni *CCs. These CCs were the largest in their respective databases. The difficulty in identifying SNPs diagnostic for the larger CCs was investigated further and was found to be due to CC members that have diverged from the CC founders by recombination rather than mutation. *S. aureus *exhibits a low recombination frequency, with approximately 10% of CC members arising by recombination [27]. While mutation predominantly generates single novel SNPs, recombinants acquire a pre-existing allele from elsewhere in the species. The large pool of alleles present in the larger CCs increases the probability that one or more alleles per locus have arisen by recombination with a pre-existing allele. Given that most *S. aureus *SNPs are dimorphic, coupled with the effects of recombination and the small pool of available SNPs in *S. aureus *sequence data, there exist few SNPs unique to all members of a CC. Therefore, the probability of finding highly discriminatory SNP sets is low. In support of this, the Not-N algorithm was used to find sets of SNPs diagnostic for the 212 methicillin-resistant *S. aureus *(MRSA) STs in the MLST database [28]. The MRSA dataset is smaller than the complete *S. aureus *MLST database, and therefore the CCs contain correspondingly fewer recombinants. This analysis yielded 22 SNPs that delineated the ten major MRSA CCs with 100% confidence (results not shown).

In *C. jejuni*, the influence of recombination on Not-N performance is similar to *S. aureus *but is more extreme. The majority of STs in *C. jejuni *arise by recombination, at an estimated frequency of 50 times the rate of mutation [29], resulting in an even smaller probability of identifying CC-specific SNPs. SNP sets diagnostic for *S. aureus *CCs are more efficiently derived using the *D *maximization algorithm of "Minimum SNPs", while in *C. jejuni*, the high recombination rate renders the identification of small numbers of CC-specific SNPs with high discriminatory power and a low false-negative rate potentially impossible by any means. Other researchers have identified small numbers of *C. jejuni *CC SNPs characteristic of six major CCs of *C. jejuni*; ST-21, ST-45, ST-48, ST-61, ST-206 and ST-257 [14]. However, these SNPs result in a high proportion (between 17 and 54%) of false-negative STs, and may therefore be unsuitable for certain genotyping applications.

The "Minimum SNPs" software has previously been used to derive *D*-optimized (diversity maximizing) SNP sets from the *S. aureus *and *C. jejuni *databases [21-23]. In the case of *S. aureus*, the genotypes defined by approximately eight *D*-optimized SNPs correspond closely to the population structure as defined by eBURST analysis. Thus, the *D*-optimized SNP sets appear superior to the Not-N derived SNP sets for assigning an *S. aureus *isolate to a CC. In the case of *C. jejuni*, the correspondence between *D*-optimized SNP genotypes and population structure is less than in *S. aureus *because of the higher recombination frequency. However, adding the interrogation of more loci such as a hypervariable region (sequencing of the flagellin A short variable region) to the SNP-based genotyping reduced to insignificance the incidence of unrelated isolates failing to be discriminated, thus demonstrating the value of the *D*-optimized SNP set. In summary, for both species, Not-N-derived SNPs are generally not optimal for assigning isolates to larger CCs, but are highly effective for identifying the smaller CCs.

### CGH datasets

CGH allows the large-scale identification of genetic differences across a number of strains. Bayesian-based algorithms applied to the CGH data of *C. jejuni*, *Y. enterocolitica *and *C. difficile *enabled the identification of phylogenetic clades that can predict infection source or pathogenicity traits. Champion et al. [5] identified two distinct clades in *C. jejuni *predictive of infection source, with one clade containing predominantly livestock isolates and the other non-livestock (environmental) isolates. Isolates identified from human infection were roughly evenly distributed between the two clades. In *Y. enterocolitica*, three clades corresponding to non-pathogenic (biotype 1A), low-pathogenicity (biotypes 2–5) and highly pathogenic (biotype 1B) were identified by the comparative phylogenomics approach [4]. For *C. difficile*, CGH phylogeny identified four clades comprising a hypervirulent clade, a toxin A-B+ clade, and two clades containing human and animal isolates [6].

The three CGH studies identified binary genes specific to particular clades using MacClade, parsimony-based software that is used for reconstructing phylogeny and interpreting patterns of character evolution [30]. MacClade 4 was used to identify 33 coding sequences (CDS) in *C. jejuni *that were characteristic of the livestock clade, including the gene cluster *Cj1321 *to *Cj1326 *within the O-linked flagellin glycosylation locus. However, none of the 33 CDS identified by MacClade 4 analysis were 100% specific to the livestock clade [5]. In contrast, Not-N analysis identified two binary genes from the *C. jejuni *CGH data that separated, with 100% confidence, isolates from the livestock clade from those in the nonlivestock clade (Table 1).

**Table 1:** Not-N analysis-derived binary gene targets from CGH data of *Campylobacter jejuni*, *Yersinia enterocolitica *and *Clostridium difficile*.

**Bacterium**	**Clade^a^**	**Gene 1 (%)**	**Gene 2 (%)**	**Gene 3 (%)**	**No. of Pathways^b^**
*C. jejuni*	Livestock	*Cj0818*, present (85.7)	*Cj0424*, present (100)	---	8
	Non-livestock	---	---	---	n/a
*Y. enterocolitica*	Non-pathogenic	Ye8081-4002, absent (100)	---	---	0
	Low pathogenicity	Ye8081-0306, absent (100)	---	---	15
	Highly pathogenic	Ye8081-0113, present (100)	---	---	50
*C. difficile*	HY	CD2669, absent (98.1)	CD2983, present (100)	---	13
	A-B+	CD2983, absent (100)	---	---	0
	HA1	CD2570, present (46.4)	CD2669, present (83.9)	CD2983, present (100)	0
	HA2	CD0265, absent (100)	---	---	8

^a ^Clades defined for *Y. enterocolitica*,*C. jejuni *and *C. difficile *by references [4-6] respectively.

^b ^Corresponds to the number of alternate outputs provided by Not-N analysis that are not shown in the. n/a, not applicable.

Within the *Y. enterocolitica *CGH data, MacClade 4 analysis identified several CDS that were 100% specific to each of the three pathogenicity clades [4]. Some of these genes, such as YE1820 (characteristic of the non-pathogenic clade), were classed as divergent based on the array signal but considered present for the purposes of the MacClade analysis. CGH cannot reliably detect small differences in hybridizations caused by moderate gene divergence [31]. For this reason, only genes that were clearly present or absent were analyzed by Not-N to remove potential miscalled binary gene status. Three binary genes were identified that enabled 100% discrimination between the differing pathogeneses of *Y. enterocolitica*. In *C. difficile*, MacClade analysis did not identify binary gene sets specific for isolates within the four clades, and many of the identified genes were divergent [6]. In comparison, Not-N analysis identified four binary genes that separated the four distinct clades described by Stabler et al. [6] with 100% confidence. These results demonstrate that the application of Not-N to CGH data was both more efficient and able to identify fewer binary targets than MacClade analyses (Table 1).

### Viral sequence datasets

A large number of complete genome sequences are currently available for both HCV (188 genomes) and HIV-1 (1507 genomes) [8,10], providing an ideal resource to examine the performance of Not-N analysis on well-characterized loci within these viruses. Despite examining several loci, Not-N analysis was unable to identify SNPs diagnostic for any of the HIV-1 clades. This is likely due to the exceptionally high degree of recombination between HIV-1 variants that has resulted in the emergence of circulating recombinant forms (CRFs) [32]. Given these results, and the acceptance of sequencing as the appropriate HIV-1 genotyping approach, it was concluded that Not-N derived SNPs are not suitable for HIV-1 genotyping. In contrast, Not-N analysis identified 15 HCV SNPs that delineate, with 100% confidence, the 13 predominant subtypes of this virus (Table 2). Interestingly, Not-N analysis failed to identify comparably informative SNP sets for the six major genotype groups (1–6) of HCV, possibly as a consequence of the high level of divergence between subtypes within each genotype group.

**Table 2:** Single-nucleotide polymorphisms identified by Not-N analysis for the major subtypes of hepatitis C virus.

**HCV subtype**^a^	**No. genotypes**	**SNP 1**		**SNP 2**	
		
		Position^b^	Discrimination (%)	Position	Discrimination (%)
1a	117	126* (C or T)	100	-	-
1b	382	103 (C)	72.1	194* (G)/238* (C) 100%	100
2a	38	258 (A or G) 99.7%	99.7	182* (T)	100
2b	53	314 (A)	99.7	127 (A)	100
2c	5	50 (T)	99.7	39* (G)	100
3a	49	295 (G)	100	-	-
3b	6	307 (T)	99.8	126* (G)	100
4a	26	182* (A)	97.8	325* (A)	100
4d	17	154 (A)	100	-	-
4f	21	325* (C)	99.5	194* (T)/238* (A) 100%	100
4t	4	338/339 (C)	100	-	-
5a	18	100 (C)	100	-	-
6a	34	39* (C or T)	100	-	-

^a^Subtypes containing less than four confirmed sequences were not included in the analysis. Sequences were downloaded from the hepatitis C virus (HCV) sequence database [10].

^b^The single-nucleotide polymorphism (SNP) position refers to a 340 bp fragment of the RNA-dependent RNA polymerase NS5B spanning nucleotides 8276 to 8615 (GenBank accession AF009606 [48]). NS5B is used to construct phylogenetic trees for HCV, which form the basis of the genotype and subtype nomenclature [40].

*SNP discriminates multiple subtypes.

Current HCV genotyping methods such as the line probe assay (INNO-LiPA) [33], the real-time PCR-based Abbott HCV analyte-specific reagent (ASR) and COBAS TaqMan48 HCV tests [34,35], restriction fragment length polymorphism analysis [36] and primer extension methods [37] primarily target the 5' non-coding region (5'-NCR). A drawback of targeting the 5'-NCR is that some subtypes, such as 1a and 1b, 1b and 6a, or 2a and 2c, remain indistinguishable in a small number of cases due to the conserved nature of this region [38,39]. In contrast, the 15 HCV SNPs identified by Not-N analysis were derived from RNA-polymerase NS5B rather than the 5'NCR, and unlike 5'-NCR-derived SNPs, are 100% specific for each of the 13 subtypes of HCV. This finding is significant as the correlation between HCV genotype and clinical outcome is well-documented. Genotype-specific differences between HCV variants aid in assessing the clinical management of infection, with genotypes 1 and 4 more resistant than genotypes 2 and 3 to interferon-α-based therapy. In addition, HCV variants appear specific to particular geographic regions, such as the widespread distribution of HCV subtype 1a throughout the USA and Northern Europe [40].

To our knowledge, this is the first set of genotyping targets that enables the specific and accurate discrimination of the 13 major subtypes of HCV. Real-time PCR-based methods, such as allele-specific real-time PCR [41] or high-resolution melt analysis [42], are promising candidates for interrogating the 15 SNPs due to their ability to accurately interrogate polymorphisms in diverse DNA sequence, such as that found within the NS5B region of HCV.

## Conclusion

This study has shown that the Not-N algorithm provides a practical tool for identifying diagnostic polymorphisms that discriminate bacterial or viral populations of interest. Not-N analysis was particularly valuable with bacterial CGH and HCV genome sequence data, where the software identified genetic markers with superior performance to polymorphisms in current use. The ability of the algorithm to select SNPs diagnostic for MLST-defined CCs was dependent on CC size, with large numbers of SNPs required to delineate the larger CCs that have undergone extensive recombination. The purpose of the Not-N algorithm is conceptually similar to the identification of canonical phylogenetic SNPs, such as those previously described for *Bacillus anthracis *[43], and indeed would be valuable for identifying canonical SNPs in other clonal populations. Not-N analysis is likely to become increasingly useful as comparative databases expand in size, and as more is uncovered about the relationships between pathogen genotype, infection epidemiology and clinical outcomes. This approach to data analysis may also be applied to the identification of discriminatory sets of genetic polymorphisms that have direct biological significance, rather than being simply diagnostic markers. In such instances, it may not be so critical for the "0% false negative" criterion to be fulfilled. This allows an approach in which the analysis is carried out twice, with one or other of the two groups of variants defined as the "group of interest" in each case. This increases the probability that informative sets of polymorphisms will be identified.

## Methods

### Bacterial and viral databases

The sequence type (ST) and allele files for *C. jejuni*, *S. aureus*, and *Haemophilus influenzae *were downloaded from the MLST databases for these organisms [28]. For *Escherichia coli*, data was obtained from two MLST schemes using seven loci [44-46], eBURST v3 was used to assign STs to clonal complexes (CCs) [47]. For *S. aureus *and *C. jejuni*, CCs were defined as STs sharing 6/7 loci with the ancestral clone, whereas with *H. influenzae *and *E. coli*, this parameter was set to 5/7 loci and 4/7 loci, respectively.

Sequence data for HIV-1 and HCV were downloaded from the respective databases [8,10]. The region spanning nucleotides 8276 to 8615 of the HCV genome corresponding to a partial sequence of the RNA-dependent RNA polymerase, NS5B, was chosen for SNP analysis as this region is used to construct phylogenetic trees for HCV [40]. In total 770 NS5B sequences were analyzed. The thirteen confirmed HCV genotypes were examined: 1a, 1b, 2a, 2b, 2c, 3a, 3b, 4a, 4d, 4f, 4t, 5a and 6a. For HIV-1, we tested the ability of the Not-N algorithm to select SNPs that would identify the genotype M group as this genotype comprises over 99% of human HIV-1 infections [32].

CGH array data for *C. jejuni*, *Y. enterocolitica *and *C. difficile *was downloaded from BμG@s [48] accessions E-BUGS-22, E-BUGS-36 and E-BUGS-41). CGH data was filtered to exclude genes considered divergent in one or more strains, and 'flagged' genes (data missing in one or more strains, due to a poor array signal). Based on these criteria, 696, 1080 and 785 genes from the available 111 *C. jejuni*, 93 *Y. enterocolitica *and 74 *C. difficile *strains [4-6] were analyzed using the Not-N module of "Minimum SNPs". Gene presence or absence was converted to nucleotide format to enable "Minimum SNPs" analysis as previously described [24]. Isolates were grouped for Not-N analysis according to previous CGH phylogeny with the exception of *Y. enterocolitica *strain 237_02, which was shown to group with the non-pathogenic clade following ClustalX phylogenetic analysis [49] and visualization using TreeView 1.6.6 [50] of the filtered dataset.

### The Not-N algorithm and its implementation

The Not-N algorithm is designed to derive, from sequence alignments, sets of SNPs or binary genes that discriminate a user-defined subset of the isolates (the group of interest) from all the other genotypes in the alignment (the out-group). The fundamental principle of this algorithm is that it does not treat the group of interest and the out-group equally. A position in the alignment is only considered informative if one or more bases that are present at that position in the out-group are not present in any of the sequences in the group of interest. The resolving power of a position is the proportion of out-group sequences that contain the base(s) in common with the group of interest. The rationale for the algorithm design is twofold. Firstly, the derived SNP sets cannot give rise to false-negatives since SNPs are specifically selected to identify all members of a group of interest. This lack of false-negatives is important if the SNP sets are to form the bases of e.g. diagnostic procedures for identifying virulent subgroups within a species. Secondly, Not-N can accommodate polymorphisms within the group of interest; that is, the algorithm is not reliant on identifying only invariant sites within the group of interest. Therefore, Not-N efficiently uses the available sequence data. The algorithm is demonstrated in Table 3. STs 1, 2, and 3 are the group of interest whilst the remaining STs are the out-group. A consensus sequence is assembled by scoring each nucleotide in the alignment as 'Not-A/C/G/T'. The out-group sequences are subsequently scored as a match (+) or mismatch (-) relative to the consensus sequence for the group of interest.

**Table 3:** The Not-N algorithm and its implementation by the Minimum SNPs computer program. A. Data for seven hypothetical sequence types (STs) at six single-nucleotide polymorphisms (SNPs). B. Not-N analysis output of the alignment at A. Four sets of two SNPs are identified, all of which reach 100% discrimination. C. Result obtained if positions 3 and 4 are excluded.

A.						
Sequence ID	SNP 1	SNP 2	SNP 3	SNP 4	SNP 5	SNP 6

ST 1*	G	G	G	A	T	T
ST 2*	A	G	T	T	T	G
ST 3*	G	G	G	C	T	G
ST 4	A	G	A	G	A	T
ST 5	A	A	A	G	T	T
ST 6	A	G	T	C	A	A
ST 7	A	A	A	G	T	G

Consensus (STs 1, 2 & 3)	Not informative	Not-ACT	Not-AC	Not-G	Not-ACG	Not-AC

>ST4	n/a	+	-	-	-	+
>ST5	n/a	-	-	-	+	+
>ST6	n/a	+	+	+	-	-
>ST7	n/a	-	-	-	+	+

Confidence (%)	Position not used	50	75	75	50	25

*"Group of interest" sequences						

B.						

SNP set	SNP 1 position and consensus	Cumulative discriminatory power (%)	SNP 2 position and consensus	Cumulative discriminatory power		

1	3, NOT AC,	75	5, NOT ACG	100		
2	3, NOT AC	75	6, NOT AC	100		
3	4, NOT G	75	5, NOT ACG	100		
4	4, NOT G	75	6, NOT AC	100		

C.						

SNP set	SNP 1 position and consensus	Cumulative discriminatory power (%)	SNP 2 position and consensus	Cumulative discriminatory power (%)		

1	2, NOT ACT,	50	5, NOT ACG	100		

The Not-N function has been incorporated into the "Minimum SNPs" version 2.043 software [19,23]. Previous versions of this software identified SNP sets on the basis of two user-selectable performance criteria:maximization of *D*, or maximization of the power to discriminate one user-selected sequence from all known sequences. The Not-N algorithm represents a third user-selectable performance criterion for SNP set assembly. The SNP sets are assembled one SNP at a time, with the SNP giving the highest informative power identified first, followed by the SNP that gives the highest informative power in combination with the previous SNP, and so forth. Where different SNPs have identical informative power, multiple SNP combinations are assembled, until either a pre-set level of discrimination, a pre-set number of SNPs, or 100% discrimination is reached. The software incorporates 'include' and 'exclude' functions that allow the operator to force the inclusion of one or more SNPs in the output SNP set, or to remove one or more SNPs from the analysis. This provides considerable flexibility in SNP set assembly, which can be of benefit when optimizing actual assays, and provides a means of protecting against "local minima" (SNP sets with non-optimal resolving power due to pathway constraints imposed by the first identified SNP). In the example shown in Table 3, SNP 1 is classed as non-informative as the group of interest is not deficient in any bases in comparison to the out-group at that position, whilst SNPs 2 to 7, either alone or in combination, discriminate the group of interest from the out-group with different levels of confidence. Use of the exclude function to remove SNPs 3 and 4 yields a new SNP set that reaches 100% discrimination (Table 3C) as efficiently as the SNP sets in Table 3B.

## Availability and Requirements

"Minimum SNPs" version 2.043, together with documentation, may be obtained from . There is a requirement to agree to a click-wrap license that is applicable to non-commercial use only. The software is written using the Java Runtime Environment which makes is essentially platform independent. Users need to have the Java Runtime Environment installed on their computer. This is freeware that can be obtained from . Downloading of this also requires agreeing to a license.

## Competing interests

The authors on this manuscript are inventors on patent applications describing this algorithm and its applications. They may in consequence be eligible for financial benefit if these patent applications are commercialised.

## Authors' contributions

EPP carried out most of the data analysis and drafted the manuscript. JI-B and FH carried out additional data analysis. VT wrote the source code of the Not-N algorithm for integration into the "Minimum SNPs" software. PG conceived of the study, and participated in its design and coordination and helped draft the manuscript. All authors read and approved the final manuscript.

## Supplementary Material

Additional file 1 Single-nucleotide polymorphisms extracted from multilocus sequence typing data using the Not-N module of "Minimum SNPs". Click here for file
