# Supplementary material for: Computer-aided identification of polymorphism sets diagnostic for groups of bacterial and viral genetic variants
Source: BMC Bioinformatics. 2007 Aug 1;8:278. doi: 10.1186/1471-2105-8-278 (PMC1973086; doi:10.1186/1471-2105-8-278)
Supplement: Additional file 1 — Single-nucleotide polymorphisms extracted from multilocus sequence typing data using the Not-N module of "Minimum SNPs". [file 1471-2105-8-278-S1.doc]

**Single-nucleotide polymorphisms extracted from multilocus sequence typing data using the Not-N module of “Minimum SNPs”.**

| CC | No. STs | SNP 1 (%) | SNP 2 (%) | SNP 3 (%) | SNP 4 (%) | SNP 5 (%) | SNP 6 (%) | SNP 7 (%) | SNP 8 (%) | No. pathways^a^ |
| --- | --- | --- | --- | --- | --- | --- | --- | --- | --- | --- |
| *Escherichia coli,* MLST scheme 1 | | | | | | | | | | |
| 1 | 105 | *lysP*198 C (71.2) | *fadD*195 A (82.1) | *uidA*518 G (93.1) | *aspC*333 A (98.6) | *aspC*131 G (100) | --- | --- | --- | 0 |
| 2 | 21 | *fadD*45 G (100) | --- | --- | --- | --- | --- | --- | --- | 26 |
| 3 | 10 | *aspA*240 C (100) | --- | --- | --- | --- | --- | --- | --- | 2 |
| 4 | 9 | *clpX*495 A (100) | --- | --- | --- | --- | --- | --- | --- | 2 |
| 5 | 8 | *uidA*518 A (100) | --- | --- | --- | --- | --- | --- | --- | 0 |
| 6 | 6 | *icdA*126 T (98.2) | *icdA*336* G (100) | --- | --- | --- | --- | --- | --- | 13 |
| 7 | 6 | *aspA*57 A (100) | --- | --- | --- | --- | --- | --- | --- | 3 |
| 8 | 5 | *mdh*291 T (75.7) | *mdh*459 G (99.4) | *icdA*225* T (100) | --- | --- | --- | --- | --- | 22 |
| 9 | 4 | *icdA*336* A (100) | --- | --- | --- | --- | --- | --- | --- | 1 |
| 10 | 4 | *icd*A225* G (100) | --- | --- | --- | --- | --- | --- | --- | 8 |
|  |  |  |  |  |  |  |  |  |  |  |
|  |  |  |  |  |  |  |  |  |  |  |
| *E. coli,* MLST scheme 2 | | | | | | | | | | |
| 1 | 270 | *fumC*416 C (52.8) | *icd*265 T (74.6) | *recA*163 C (84.7) | *fumC*257 G (90.5) | *icd*146 G (94.2) | *fumC*123 G (96.3) | *fumC*65 G (97.8) | *fumC*296 A (98.5) | 0 |
| 2 | 36 | *gyrB*180 T (92.7) | *purA*83 G (96.2) | *adk*203 A (98.6) | *adk*118* A (99.4) | *fumC*107 C (100) | --- | --- | --- | 1 |
| 3 | 23 | *mdh*348 C (98.7) | *adk*328 C (99.7) | *adk*148 C (100) | --- | --- | --- | --- | --- | 2 |
| 4 | 13 | *adk*203 T (100) | --- | --- | --- | --- | --- | --- | --- | 0 |
| 5 | 11 | *icd*331 A (100) | --- | --- | --- | --- | --- | --- | --- | 0 |
| 6 | 11 | *recA*136 C (96.9) | *mdh*85 T (100) | --- | --- | --- | --- | --- | --- | 4 |
| 7 | 9 | *recA*100 G/T (94.9) | *gyrB*372 T (100) | --- | --- | --- | --- | --- | --- | 0 |
| 8 | 9 | *fumC*107* T (100) | --- | --- | --- | --- | --- | --- | --- | 0 |
| 9 | 7 | *icd*283 T (99.5) | *adk*331 C (100) | --- | --- | --- | --- | --- | --- | 2 |
| 10 | 7 | *mdh*92 A (100) | --- | --- | --- | --- | --- | --- | --- | 0 |
| 11 | 6 | *fumC*123 T (100) | --- | --- | --- | --- | --- | --- | --- | 0 |
| 12 | 6 | *adk*118* G (100) | --- | --- | --- | --- | --- | --- | --- | 0 |
|  |  |  |  |  |  |  |  |  |  |  |
| *Staphylococcus aureus* MLST | | | | | | | | | | |
| ST-5 | 122 | n/a | n/a | n/a | n/a | n/a | n/a | n/a | n/a | n/a |
| ST-8 | 108 | n/a | n/a | n/a | n/a | n/a | n/a | n/a | n/a | n/a |
| ST-30 | 92 | n/a | n/a | n/a | n/a | n/a | n/a | n/a | n/a | n/a |
| ST-45 | 46 | *pta*312 A (99.4) | *yqiL*303* A (99.8) | *glpF*66 C (100) | --- | --- | --- | --- | --- | 23 |
| ST-1 | 46 | n/a | n/a | n/a | n/a | n/a | n/a | n/a | n/a | n/a |
| ST-97 | 35 | *aroE*212 A (87.4) | *yqiL*303* A (97.6) | *glpF*276* G (99.2) | *arcC*78 G (99.6) | *pta*85* G (99.8) | --- | --- | --- | 0 |
| ST-15 | 35 | *arcC*199 A (59.5) | *yqiL*333 T (85.6) | *pta*85* G (97.4) | *pta*294 A (98.9) | *aroE*238 G (99.0) | *gmk*16 C (99.2) | *yqiL*513 G (99.4) | --- | 0 |
| ST-121 | 27 | *arcC*184 A (95.5) | *aroE*102 T (99.1) | *pta*85* A (100) | --- | --- | --- | --- | --- | 8 |
| ST-22 | 21 | *yqiL*168 A (95.9) | *yqiL*88 G (99.8) | *pta*85* A (100) | --- | --- | --- | --- | --- | 39 |
| ST-133 | 18 | *aroE*79 G (100) | --- | --- | --- | --- | --- | --- | --- | 4 |
| ST-78 | 14 | *glpF*231 C (100) | --- | --- | --- | --- | --- | --- | --- | 1 |
| ST-59 | 13 | *pta*177 G (100) | --- | --- | --- | --- | --- | --- | --- | 1 |
| ST25 | 9 | *glpF*276* A (100) | --- | --- | --- | --- | --- | --- | --- | 1 |
|  |  |  |  |  |  |  |  |  |  |  |
| *Campylobacter jejuni* MLST | | | | | | | | | | |
| ST-21 | 422 | n/a | n/a | n/a | n/a | n/a | n/a | n/a | n/a | n/a |
| ST-825 | 400 | *glyA*42 A/C (68.1) | *glyA*3* T (92.7) | *glnA*45 G/T (97.4) | *glnA*108* G (98.2) | *tkt*189* A/G/C (98.7) | *glnA*240 A (98.9) | *tkt*28 T (99.1) | *glnA*132* A (99.2) | 0 |
| ST-45 | 137 | n/a | n/a | n/a | n/a | n/a | n/a | n/a | n/a | n/a |
| ST-257 | 67 | n/a | n/a | n/a | n/a | n/a | n/a | n/a | n/a | n/a |
| ST-353 | 57 | n/a | n/a | n/a | n/a | n/a | n/a | n/a | n/a | n/a |
| ST-177 | 50 | *gltA*180 C (99.9) | *tkt*189* T (100) | --- | --- | --- | --- | --- | --- | 18 |
| ST-42 | 33 | n/a | n/a | n/a | n/a | n/a | n/a | n/a | n/a | n/a |
| ST-403 | 31 | *tkt*234 A (90.1) | *aspA*9 C (99.3) | *aspA*342 C (100) | --- | --- | --- | --- | --- | 1 |
| ST-51 | 30 | *uncA*165 C (99.1) | *glyA*264 T (99.7) | *glnA*108* G (99.9) | *glnA*288 C (100) | --- | --- | --- | --- | 67 |
| ST-354 | 28 | *aspA*414 C (64.3) | *aspA*84 G (90.6) | *tkt*189* A/C (94.9) | *glyA*3* T (96.6) | *pgm*34 C (97.9) | *pgm*405* T (98.6) | *uncA*375* C (99.1) | --- | 0 |
| ST-52 | 26 | *glyA*504 T (79.5) | *glyA*3* T (96.7) | *uncA*375* C (98.3) | *uncA*189* C (98.9) | *glnA*12 G (99.1) | --- | --- | --- | 0 |
| ST-574 | 26 | n/a | n/a | n/a | n/a | n/a | n/a | n/a | n/a | n/a |
| ST-22 | 24 | *glyA*114 C (87.9) | *gltA*294 C (98.2) | *glnA*132* A (99.0) | *uncA*189* C (99.6) | *pgm*435 T (99.9) | *pgm*405* T (100) | --- | --- | 3 |
| ST-460 | 23 | *glnA*18 C (83.9) | *tkt*132 T (99.8) | *tkt*189* C (100) | --- | --- | --- | --- | --- | 65 |

A total of 15 single-nucleotide polymorphisms (SNPs) required to differentiate the 10 main clonal complexes (CCs) of *E. coli* (scheme 1); 24 SNPs required to differentiate the 12 main CCs of *E. coli* (scheme 2); 30 SNPs required to differentiate 9 of the 13 main CCs of *S. aureus*; and 27 SNPs required to differentiate 5 of the 13 major CCs of *C. jejuni*.

^a^ Corresponds to the number of alternate outputs provided by Not-N analysis that are not shown in the table.

*SNP discriminates multiple CCs

Fields marked ‘n/a’ failed to yield a confidence of ≥98% after eight SNPs.
